# Genetic Dissection and Germplasm Selection of the Low Crude Fiber Component in *Brassica napus* L. Shoots

**DOI:** 10.3390/foods12020403

**Published:** 2023-01-14

**Authors:** Rui Shi, Chengke Pang, Xu Wu, Xiaozhen Zhao, Feng Chen, Wei Zhang, Chengming Sun, Sanxiong Fu, Maolong Hu, Jiefu Zhang, Xiaodong Wang

**Affiliations:** 1 State Key Laboratory of Crop Genetics and Germplasm Enhancement, Nanjing Agricultural University, Nanjing 210095, China; 2 Institute of Industrial Crops, Jiangsu Academy of Agricultural Sciences, Key Laboratory of Cotton and Rapeseed, Ministry of Agriculture and Rural Affairs, Nanjing 210014, China

**Keywords:** *Brassica napus*, crude fiber, germplasm selection, QTL mapping, shoots

## Abstract

Background: *Brassica napus* is one of the most important oil crops in the world, and *B. napus* shoots are nutrient-rich fresh vegetables. The crude fiber (CF) component is one of the most important factors affecting the taste quality of *B. napus* shoots, but the factors underlying the desirable low-CF trait remain poorly understood. Methods: In this study, a high-density single-nucleotide polymorphism (SNP) map was used to map quantitative trait loci (QTLs) for five CF-related traits in a recombinant inbred population. Results: A total of 49 QTLs were obtained in four environments, including eleven, twelve, eight, twelve and six QTLs for content of neutral detergent fiber, acid detergent fiber, acid detergent lignin, hemicellulose and cellulose, respectively. The phenotypic variation explained by single QTL ranged from 4.62% to 14.76%. Eight of these QTLs were further integrated into four unique QTLs, which controlled two different traits simultaneously. Five CF-component-related candidate genes were identified, among which *BnaC03g07110D* and *BnaC07g21271D* were considered to be the most likely candidate genes. In addition, five lines with low CF content were selected, which can be used as excellent germplasm resources in breeding. Conclusions: The QTLs identified in this study will contribute to our understanding of the genetic mechanism of CF and can be used as targets for reducing CF content in *B. napus* shoots. In addition, this study also provided excellent germplasm resources for low CF content breeding.

## 1. Introduction

*Brassica napus* (2n = 38, AACC), oilseed rape, is one of the major oilseed crops in the world, with a yield of approximately 75 million tons in recent years, accounting for 13% of worldwide vegetable oil production, second only to oil palm (35%) and soybean (28%) [1,2]. In recent years, the widely cultivated double-low (low erucic acid and low glucosinolate) oilseed rape has provided healthy and nutritionally balanced edible oil for humans and protein-rich feed for animals [3]. To improve the economic value of oilseed rape, uses of this crop as vegetables, flowers, forage, honey, and fertilizer have been explored [2,4]. Oilseed-vegetable dual-purpose (OVDP) is one of the most typical, multi-functional uses, which can meet the large demand for edible oil and vegetables. In addition, OVDP can solve the problem of the “hungry gap” for fresh vegetables in the winter and spring seasons, and also alleviate the conflict of competition between land for vegetable and oil production [5]. Therefore, OVDP has an important role in enhancing the benefits of oilseed rape cultivation and promoting the development of the oilseed rape industry.

*B. napus* is an allotetraploid interspecific hybrid between *Brassica rapa* (2n = 20, AA) and *Brassica oleracea* (2n = 18, CC) in the Brassicaceae family [6,7]. Brassicas are among the most economically and nutritionally important vegetable genera, and include crops such as broccoli (*Brassica oleracea* L. var. *italic* Planch.), kale (*Brassica oleracea* var. *acephala* DC.), cauliflower (*Brassica oleracea* L. var. *botrytis* L.) and Chinese cabbage (*Brassica rapa* var. *glabra* Regel) [8]. They are rich in phytochemicals and nutrients, such as glucosinolates [9], anthocyanins [10], carotenoids [11], vitamins [12] and minerals [12]. These components confer a variety of biological properties to brassicas, including antimicrobial, antifungal, antitumor, antimutational, anti-inflammatory, neuroprotective, and antioxidant activities, and these have great beneficial effects on human health, such as protecting the heart and liver, controlling blood sugar in diabetics, modulating immunity and reducing cancer risk [8,11,12,13,14]. In addition, rapeseed has the function of selenium enrichment. As an essential nutrient element for human body, selenium can improve animal immunity and fertility. At present, several varieties of rapeseed shoots rich in selenium have been cultivated [2]. Thus, the development of *B. napus* as a vegetable has a scientific basis in encouraging consumption of this health-stimulating food.

*B. napus* shoots are the main organ targeted for OVDP use. During the bolting period, the main stem and its associated side branches are harvested as fresh vegetables or processed into dehydrated vegetables [15]. In the Yangtze River basin, the largest rapeseed-producing area in China, the seedling and bolting stage of *B. napus* is mainly in the late fall and winter, a period where there are few pests and diseases, so that crop protection chemicals are rarely used, resulting in the shoots being a contaminant-free food which is high quality and nutritious [5,15]. The contents of vitamin B1, vitamin B2, vitamin C, zinc and selenium in *B. napus* shoots are higher than those of other shoots, providing a nutritious and uniquely flavorful vegetable for consumers [16,17]. Although there have been some reports of new varieties of *B. napus* shoots, most of them focus on yield, with only a very few focusing on related quality traits [18].

Insoluble dietary fiber originating from plant cell walls is also known as crude fiber (CF), and consists of the components cellulose (Cel), hemicellulose (Hem), and lignin [19]. The detergent fiber method separates the CF into three fractions used to determine the content of each CF component so as to judge the quality of animal feed: neutral detergent fiber (NDF, mainly contains cellulose, hemicellulose and lignin), acid detergent fiber (ADF, mainly contains cellulose and lignin) and acid detergent lignin (ADL, mainly contains lignin) [20]. CF is difficult to ferment and is degraded as a source of energy [21]. In addition, the increase of the cell wall thickness and the lignification of the plant tissue affect the taste of vegetables [22]. At present, *B. napus* shoots in the market have serious homogenization, high CF content, poor palatability and other defects. It is necessary to improve the taste of shoots, towards a crisp taste, and reduce the content of stem fiber. Therefore, the appropriate reduction of CF content is an overarching goal for the breeding of high-quality *B. napus* shoots.

Quantitative trait locus (QTL) mapping is a useful method for analyzing the genetic architecture of a quantitative trait. CF-related traits are complex quantitative traits controlled by multiple genes which are markedly influenced by environmental conditions [23,24]. Asekova et al. (2016) identified two QTLs for CF, six for NDF and two for ADF in soybean, using 188 recombinant inbred lines (RILs), with individual QTLs explaining phenotypic variation (PV) ranging from 5.79% to 41.72% [25]. QTLs for NDF, ADF, and ADL have also been identified in alfalfa [26], sorghum–sudangrass (*Sorghum* × *sudangrass*) [27], spring wheat [28], and maize [29,30]. In *B. napus*, Liu et al. (2013) detected a major QTL affecting seed ADL content on the A09 chromosome and a minor QTL on the C05 chromosome, with PVs values of 31.6–42.8% and 8.1–14.1% in four different environments, respectively [23]. Five QTLs for Cel content and three QTLs related to Hem content were also detected in seeds, with PVs ranging from 4.7% to 21.9% [23]. Miao et al. (2019) obtained a total of 85 QTLs for lignin, Cel and Hem content in *B. napus* seed fiber, with PVs ranging from 2.57% to 54.76% [31]. In addition, CF components were also used to identify QTLs responsible for stem fiber, strength and rot resistance in *B. napus* [32]. However, few studies have reported on CF component content in *B. napus* shoots, and as a consequence, there is little understanding of the genetic basis of CF in this important crop.

OVDP planting mode plays an important role in improving the benefit of rapeseed, promoting the development of the rapeseed industry and meeting the new demands of consumers. In this study, QTL analysis and germplasm resource screening were conducted for five traits related to CF in multiple environments. The objectives of this study are: (1) to identify QTLs associated with NDF, ADF, ADL, Cel, and Hem in multiple environments; (2) to predict candidate genes associated with stable QTLs based on resequencing information and structural variation of the two parents; (3) to select excellent germplasm resources with low CF content. This study will provide a theoretical foundation for studying the molecular mechanism of crude fiber related traits in *B. napus* shoots and breeding high-quality varieties.

## 2. Results

### 2.1. Statistical Analysis of Crude Fiber Component

**Figure 1:**
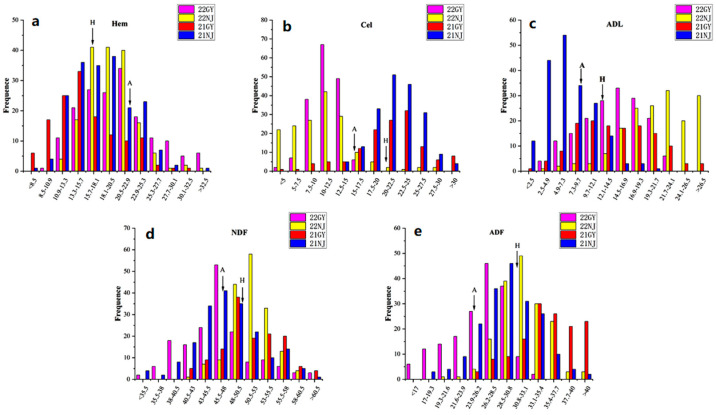
Phenotypic frequency distributions in crude fiber components of the *B. napus* shoots. The x-axis represents the content (%), and the y-axis represents the frequency. “A” and “H” indicate the average of the female parent ‘APL01′ and the male parent ‘Holly’, respectively. The codes 22GY, 22NJ, 21GY, and 21NJ are for the following different years and environments: 2022 Guiyang, 2022 Nanjing, 2021 Guiyang, and 2021 Nanjing, respectively. The frequency distribution of (**a**) hemicellulose content, (**b**) cellulose content, (**c**) acid detergent lignin content, (**d**) neutral detergent fiber content, and (**e**) acid detergent fiber content.

The CF components of the AH population and the two parents were assessed in four environments (Table 1). Except for Hem, the values for the CF-related components were higher in the male parent ‘Holly’ than that in the female parent ‘APL01′. CF components in the AH population showed extensive variation in each test environment. The maximum value in a single environment was greater than that of the higher-value parent and the minimum value was smaller than that of the lower-value parent, suggesting that synergistic alleles were distributed in both parents. The histogram showed that the frequency distribution of the CF components in the AH population conformed to normal distribution (Figure 1), indicating that CF components were typical quantitative traits controlled by multiple genes, and were suitable for QTL detection. The correlation coefficients for the CF components are shown in Table 2. Significant negative correlations were observed between NDF and Cel in Guiyang (GY), but these two components were positively correlated in Nanjing (NJ). Cel was positively correlated with both ADF and Hem in NJ, but negatively correlated with both components in GY.

### 2.2. Detecting QTLs Associated with the Five CF Traits

**Figure 2:**
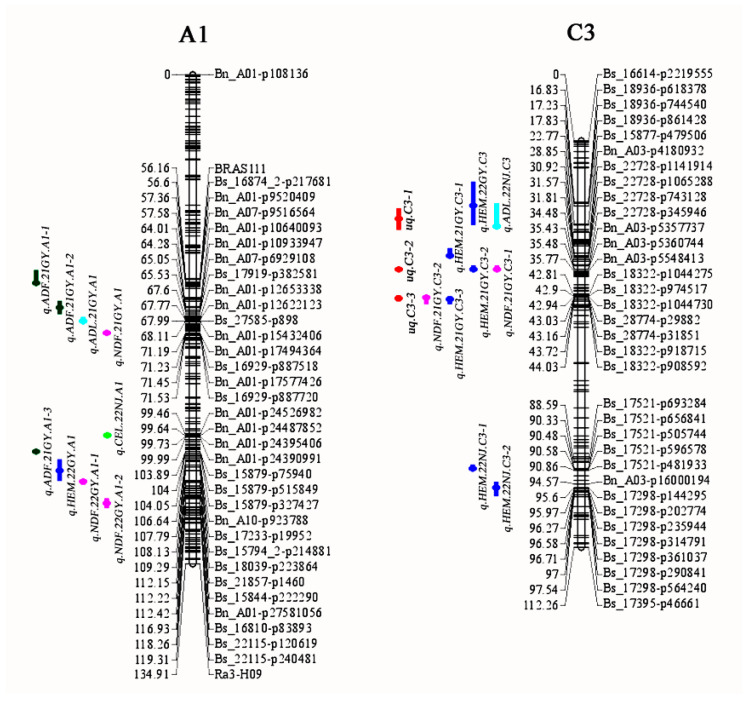
The locations of the identified and unique QTLs for crude fiber components. A1 and C3 represent chromosomes A1 and C3, respectively. The two linkage groups are represented by vertical bars. The locus names are listed on the right of the linkage groups, and the positions are shown on the left.

Based on the content of the five CF-related components in the AH population in the four environments, QTL mapping was performed using a high-density genetic linkage map. In total, 49 identified QTLs were obtained for NDF, ADF, ADL, Hem, and Cel, distributed across 14 chromosomes, with only chromosomes A3, A7, A9, C2, and C5 lacking such QTLs (Figure 2, Supplementary Table S1).

For NDF, 11 identified QTLs were detected on seven chromosomes (Supplementary Table S1). The strongest QTL, *iq.NDF.21GY.C3-1*, explained 11.49% of PV, and the APL01 allele had an additive effect of 1.6% for increased NDF. Another significant locus for NDF, *iq.NDF.21GY.C9-1*, which explained 10.38% of the PV with an additive effect of −1.53%, was located on chromosome C9. The allele from Holly at this locus was associated with an increase in NDF. The PV explained by the remaining nine QTLs ranged from 6.05% to 9.7%.

For ADF, a total of 12 QTLs located on five chromosomes was discovered (Supplementary Table S1). Among them, nine QTLs were detected in the 21GY and 21NJ environments. A QTL cluster on chromosome C6 consisted of three adjacent QTLs that explained 7.8%, 7.46% and 6.62% of the PV, respectively, and all of them had positive additive effects, suggesting that the positive alleles from ‘APL01′ contributed to the increase in ADF at the three adjacent loci.

For ADL, eight QTLs positioned on chromosomes A1, A5, C3, C4, C7 and C8 were detected, and explained the PV over a range of 4.62–9.9%, which had additive effects ranging from –2.18 to 1.61 (Supplementary Table S1). Among them, four QTLs had positive additive effects, suggesting that the positive alleles from ‘APL01′ contributed to increases in ADL. Three QTLs were located on chromosome C7 (*iq.ADL.22GY.C7*, *iq.ADL.21NJ.C7-1* and *iq.ADL.21NJ.C7-2*), including two QTLs which were detected in 21NJ and one in the 22GY environment.

For Hem content, 12 QTLs were detected on chromosomes A1, A2, C3, and C7 (Supplementary Table S1). Among them, *iq.HEM.21GY.C3-2*, with the largest PV of 14.76%, was located on chromosome C3. The ‘APL01′ allele at this locus contributed an additive effect of 2.02%. Another important QTL, *iq.HEM.21GY.C3-1*, which explained 14.16% of the PV with an additive effect of 1.95%, was also located on chromosome C3. Moreover, the QTL *iq.HEM.22NJ.A2-2* explained 13.52% of the PV, and was obtained on chromosome A2. The allele from ‘APL01′ at this locus was associated with an increase in ADF.

Six QTLs associated with Cel content were identified, which explained 5.23–9.13% of the PV (Supplementary Table S1). These QTLs were distributed unequally on different chromosomes, whereas chromosome C3 accounted for half of the total number of the Cel-associated QTLs. ‘APL01′ alleles at three loci, *iq.CEL.22NJ.A1*, *iq.CEL.21NJ.C7* and *iq.CEL.22NJ.C8*, contributed to increasing Cel content. All six QTLs were detected in NJ, with 22NJ detecting five of the QTLs for Cel.

### 2.3. Unique QTLs for the Five Traits

For the five CF-related traits, eight identified QTLs detected in different environments for different traits showed overlapping confidence intervals (CIs), suggested that these QTLs might be tightly linked QTLs in the same region or a single QTL with a pleiotropic effect on different traits (Table 3).

The QTLs *iq.HEM.22GY.13* and *iq.ADL.22NJ.13* had overlapping CIs on chromosome C3, and they were integrated into a unique QTL, *uq.C3-1*, by meta-analysis (Table 3). The unique QTL *uq.C3-1* had different favorable allele contributions, with positive additive effects on HEM in the 22GY environment, but negative additive effects on ADL in the 22NJ environment.

Two identified QTLs (*iq.NDF.21GY.13-1* and *iq.HEM.21GY.13-2*) were integrated into one unique QTL, *uq.C3-2*, while *uq.C3-3* was integrated from *iq.NDF.21GY.13-2* and *iq.HEM.21GY.13-3* (Table 3). These two unique QTLs simultaneously affected both NDF and Hem, and had positive additive effects, a finding which was consistent with the highly significant positive correlation between NDF and Hem in the GY environment. Likewise, *uq.C7* was also associated with both ADL and Hem, with negative additive effects on both CF components. These four unique QTLs represented promising genomic regions for investigation to improve CF by marker-assisted selection.

### 2.4. Candidate Gene Prediction

Four unique QTLs simultaneously affected different traits, indicating that important genes controlling the CF composition of *B. napus* shoots might exist in these QTL intervals (Table 3). QTLs *uq.C3-1*, *uq.C3-2* and *uq.C3-3* located on chromosome C3 corresponded to the intervals 18.63–24.3 Mb, 34.95–35.86 Mb and 42.75–44.16 Mb on the physical map, whereas *uq. C7* correspondeds to the intervals 48.18–51.63 Mb on the physical map on chromosome C7. According to the resequencing results of ‘Holly’ and ‘APL01′, corresponding genes and structural variations therein were screened for within the corresponding intervals of the four QTLs, and a total of 349 candidate genes were identified. The 349 genes were targeted and candidate genes related to CF components of *B. napus* shoots were identified by functional annotation of *Arabidopsis* homologous genes.

Within the CI of QTL *uq.C3-1*, two candidate genes were screened, namely *BnaC03g07110D* homologous with *AT5G60920* and *BnaC03g09000D* homologous with *AT5G18410* (Table 4). Functional annotation of the *BnaC03g07110D* homolog showed that it is involved in cell expansion and cellulose biosynthesis in the plant cell wall [33]. *AT5G60920* was also identified as a potential regulator of cellulose biosynthesis and was primarily associated with microfiber deposition during rapid elongation [34,35]. In addition, *AT5G18410* has been associated with actin cytoskeleton organization [36]. At the same time, resequencing results showed structural variation in these two genes, so that they are presumed to be candidate genes controlling CF components.

The *Arabidopsis* homolog, *AT3G61610*, of the candidate gene, *BnaC03g12990D*, which was included in the CI of *uq.C3-2*, encoded a member of the galactose mutase-like superfamily of proteins involved in carbohydrate metabolism (Table 4). Previous studies have shown that cell wall mutants in *Arabidopsis* have certain effects on secondary cell wall composition [37]. CF is a non-starch polysaccharide in carbohydrate and a component of the cell wall, so it is speculated that this gene is a candidate gene related to a CF component. The interval of QTL *uq.C3-3* partly overlaps with QTL *uq.C3-2*, and the same candidate genes were found.

Within the CI of *uq.C7*, two candidate genes, *BnaC07g21271D* (homolog of *AT1G14720*) and *BnaC07g21371D* (*AT1G14690*), were found (Table 4). The *AT1G14720* gene encodes a member of an enzyme family, xyloglucan endotransglucosylase/hydrolases (XTHs), that mediates the construction and restructuring of xyloglucan cross-links and which plays a key role in the construction and disassembly of cell wall structures in various plant tissues [38,39]. The homolog of *BnaC07g21370D* (*AT1G14690*) encodes a microtubule-associated protein, MAP65-1. Previous studies had shown that the MAP65-1 group of microtubule-associated proteins is responsible for the binding of cortical microtubules during secondary cell wall formation and primary cell wall expansion [40]. The *Arabidopsis* homologs of these two genes were related to cell wall formation and structure construction, and resequencing results showed that there was structural variation in each of these two genes, so they were considered to be candidate genes related to CF components.

### 2.5. Selection of Germplasm Resources

According to the phenotypic statistics, the values for each trait averaged over two years (2020–2021 and 2021–2022) and two locations (NJ and GY) were calculated, and the ten lines with the lowest content of each of CF, Cel, Hem and ADL were selected, respectively (Table 5). There were significant differences in the content of each trait between NJ and GY, and the average content of each trait in NJ was higher than that in GY, except for ADL. In the NJ location, the CF content of AH162 and AH172 were significantly lower than the average value, and AH162 was also among the ten lines with the lowest HEM and ADL content. AH172 was also one of the ten lines with the lowest ADL content, so AH162 and AH172 represent excellent germplasm resources for breeding for low-CF *B. napus* shoots in NJ and the surrounding area.

In trial site GY, the CF content in AH032 and AH018 lines were significantly lower than the average content, and the two lines were also among the ten lines with the lowest Hem content (Table 5). In addition, AH121 had the lowest ADL content and a CF content lower than average, so the three lines can be considered as valuable parental materials in the GY location. Encouragingly, AH034 was found among the ten lines with the lowest CF content at both GY and NJ locations. Therefore, AH034 has strong adaptability and can be used as an excellent germplasm resource for breeding for low CF content in *B. napus* shoots.

## 3. Discussion

Double-low *B. napus* shoots harvested at the bolting stage of growth can be used as a new type of nutritious vegetable, a development which can increase the income for growers without affecting the seed yield for oil extraction [17]. *B. napus* shoots have the familiar and attractive (to the consumer) aroma of brassicas, and are rich in vitamin C, calcium, zinc and selenium, all factors which are beneficial to human health. In addition, *B. napus* has a special selenium-rich function, which means it can be used as a high-quality functional vegetable [41]. The current market for *B. napus* shoots is currently limited by factors such as large leaves and high CF content [42]. The high CF content negatively affects the texture of the shoots, and therefore has a detrimental impact on consumer choice. Breeding *B. napus* varieties well adapted for shoot harvesting is the best way to meet the demand of consumers. However, the genetics of CF content in *B. napus* shoots is poorly understood, and superior germplasm resources are therefore very scarce. In order to analyze the genetic basis of CF-related traits in *B. napus* shoots, QTL mapping and candidate gene analysis were performed in the present study, which laid a foundation for the further study of CF related traits in shoots.

The main edible part of *B. napus* shoots is the tender stem and leaf at the bolting period. During growth, the lignin content of the cell wall increases, leading to an increase in cell wall thickness and negatively affecting the taste and texture of the shoots [22]. At present, most of the current research on CF is focused on the CF components of forage crops [27,28], lodging resistance of crop stems [32] and seed fiber [31,43]. In this study, a total of 49 QTLs were detected, with PV ranging from 4.62% to 14.76%, including five QTLs which individually explained more than 10% of PV (Supplementary Table S1). In addition, four unique QTLs simultaneously affected two different CF-associated traits and were integrated by meta-analysis (Table 3). To our knowledge, the research described here is the first QTL mapping study of CF components in *B. napus* shoots. Among the 49 QTLs, most of them were environment specific QTLs that were detected in only a single environment. There may be two reasons for this phenomenon. First, CF components are quantitative traits controlled by multiple genes, and there were no major QTLs associated with these traits. Second, CF components are easily influenced by the environment, and the environmental differences between field trial sites, such as temperature, light and rainfall, are inevitable and uncontrollable [5]. In order to confirm the results of the present study, multi-locus experiments need to be carried out over different years in the future.

Although some studies have predicted the candidate genes of CF components in other crops, few genes have previously been identified to regulate the CF components of *B. napus* shoots. In the current study, five candidate genes related to CF components were identified by screening, among which *BnaC03g07110D* and *BnaC07g21271D* were the most important candidate genes (Table 4). Cellulose is the skeleton of the primary wall, and *AT5G60920* (*COBRA*), the homolog of *BnaC03g07110D*, has been found to be involved in cell expansion and to be essential for the biosynthesis of cell wall components [44]. At present, the *COBRA* gene has been widely studied in model plants such as tomato, and its gene function has been verified, but there are few studies in rapeseed. Therefore, this will provide a basis for the study of cell wall development and CF synthesis in *B. napus* [39]. The functional annotation showed that *BnaC07g21371D* encodes a xyloglucan endotransglucosylase/hydrolase (XTH), which is involved in the extensibility or other mechanical properties of cell walls in various plant tissues [39]. The *Arabidopsis* XTH family gene *AtXTH28* has a potential regulatory role in cell wall construction and modification, and cell wall formation plays an important role in normal stamen development [38]. The main components of CF are derived from the cell wall. The functions of the *Arabidopsis* homologs *BnaC03g07110D* and *BnaC07g21271D* are related to the cell wall biosynthesis process. However, the molecular mechanism of the CF component synthesis in the cell wall of *B. napus* shoots is still unclear, and the functions of candidate genes need to be further verified in the future. The candidate genes and loci detected in the present study provide valuable information for the future understanding of the genetic mechanism of CF components in *B. napus* and other Brassica vegetables.

At present, there are few germplasm resources of *B. napus* shoots with excellent quality. In this study, two *B. napus* lines, AH162 and AH172, were found to exhibit low CF content in the NJ trial location. Three lines with low CF were also identified in GY, namely AH032, AH018 and AH121. Most importantly, AH034, a line with low CF content in both locations, was found. The differences of each trait between NJ and GY were large, and the coincidence degree of the materials with excellent traits was low, which indicated that the environment had a marked influence on these traits, a finding which was corroborated by the result that most of the QTLs for CF content were environment-specific QTLs. Based on the selected lines, if low-CF varieties were bred for local types, such as NJ in the lower reaches of the Yangtze River, the lines AH162 and AH172 could prove to be excellent parents. To breed low-CF varieties for promotion on the Yunnan–Guizhou Plateau, such as GY, the lines AH032, AH018 and AH121 should be selected as best-adapted parents. If a low-CF variety with broad adaptability is needed, AH34 could be selected as an appropriate parent. Surprisingly, lines AH162 and AH121 were also among the ten lines with the highest soluble solids content (SSC) at the NJ and GY locations, respectively [5]. The SSC can cover the bitterness and spiciness of shoots, and increase the sweetness, making the shoots more acceptable and palatable to consumers [5]. These result mean that AH162 and AH121 are breeding lines combining high SSC and low CF content. This study provided superior germplasm resources for the low fiber breeding of *B. napus* shoots.

## 4. Materials and Methods

### 4.1. Plant Material

The RIL population (named the AH population) was constructed by selecting 189 lines from 550 F_9_ RILs and was used to detect QTLs for CF components, with the apetalous line ‘APL01′ and the Canadian variety ‘Holly’ as the parents. A high-density genetic linkage map was constructed using the Brassica 60 K Infinium BeadChip Array, which contained 2755 bins involving 11,458 SNPs and 57 SSRs. The map covered 2027.53 cM, with the average distance between adjacent markers being 0.72 cM [45].

So far, the AH population has been successfully used to detected QTLs for apetalous character [45], seed fatty acid composition [46], flowering time [47], soluble solids content in *B. napus* shoots [5], and seed weight and shape [48]. In this study, the AH population was used for QTL mapping and candidate gene analysis of five CF-related trait components in *B. napus* shoots.

### 4.2. Field Trials

The 189 RILs and their parents were planted in four experiments (year × location combinations were regarded as different experiments). Materials were planted for two consecutive years (September–May of 2020–2021 and 2021–2022) in Nanjing, Jiangsu Province and Guiyang, Guizhou Province (21GY, 21NJ, 22GY, 22NJ, respectively). All experiments were conducted with two biological replications, and each line was planted in a two-row plot, with 20 plants per row and 40 cm between the rows [48].

### 4.3. Measurement of Crude Fiber Components

According to the national agricultural industry-standard “Grades and specincations of flowering Chinese cabbage”, three uniform *B. napus* shoots, each 20 cm in length, 1.5–1.8 cm in cross diameter at the cut end, with intact leaf shape, no withering and with unopened flower buds, were harvested at the bolting stage [5]. These harvested shoots were collected in an electric thermostatic blast dryer (DGG-9240A, Senxin, Shanghai, China), killed by 105 °C for 15 min, then dried at 80 °C to a constant weight. The dried shoots were then crushed individually to 1 mm particle size (by passage through an 18–mesh sieve) using a crusher and used as samples for measurement as described in GB/T 6434-2006 [49].

The contents of NDF, ADF, ADL, Hem and Cel in the *B. napus* shoots were determined using the polyester filter bag method according to the description of Zhang et al. (2015) [50]. This method is simple, convenient, rapid and accurate, allowing for rapid batch determination. After neutral detergent, acid detergent and 72% sulfuric acid treatment, the content of each component of CF can be obtained.

For NDF determination, accurately weigh about 1.0 g (m) of the sample in a polyester filter bag of known mass (m_1_), seal it with a plastic sealer, and label it, before adding 1000 mL of neutral detergent solution (3% sodium dodecyl sulfate solution) and boiling for 60 min [50]. During the boiling period, the concentration of the detergent solution is kept constant. After boiling, the polyester filter bag is removed, rinsed with water and squeezed dry. The polyester filter bag is then placed in a beaker and soaked in acetone for 10 min to remove the remaining fat, before being allowed to cool in a fume hood. The polyester filter bag is then dried in the oven at 105 °C for 1 h, taken out and weighed immediately and recorded as m_2_. The content of NDF was then calculated by Equation:

NDF% = (m_2_ − m_1_)/m × 100%

For ADF determination, put the polyester filter bag after NDF determination into 1000 mL acid detergent solution (2% cetyl trimethyl ammonium bromide solution) and boil for 60 min [50]. The subsequent steps were as described for NDF determination. After drying at 105 °C for 1 h, the polyester filter bag was taken out and weighed immediately and recorded as m^3^ [50]. ADF concentration was determined by Equation:

ADF% = (m_3_ − m_1_)/m × 100%

For ADL determination, the residue in the ADF filter bag was kneaded and put into a beaker with 12.0 mol/L sulfuric acid at 20–25 °C for 3 h, where it was broken into smaller pieces with a glass rod and stirred once every 30 min [50]. The material was then taken out and washed with water to neutralize it, the water was squeezed out and the residue was put into an oven at 105 °C for 1 h, the taken out and weighed immediately, with the weight recorded as m_4_ [50]. The content of ADL was then calculated by Equation:

ADL% = (m_4_ − m_1_)/m × 100%

The residual ash was negligibly small in this experiment. According to the principle of fiber determination by Soest et al. (1967) [51], with modifications, the content of Hem and Cel were calculated by the following Equations:

Hem (%) = NDF% − ADF%

Cel (%) = [ADF% − ADL%]/m

where m is the mass of the sample.

### 4.4. QTL Detection and Meta-Analysis

QTL analysis for Cel, Hem, NDF, ADF and ADL content of *B. napus* shoots was performed by composite interval mapping using the Windows QTL Cartographer 2.5 [45] The walking speed was set to 2 centimorgans (2 cM) and a window size of 10 cM with five background cofactors. The significance level of logarithm of odds (LOD, 2.5–2.9) for each trait was set by 1000 permutation tests (5% overall error level). The QTLs with LOD greater than the significance level were designated as identified QTLs. These identified QTLs with overlapping CIs for different traits were then further integrated into a unique QTL by meta-analysis using BioMercator 4.2 software with default parameters [52].

The nomenclature of the QTLs was based on the description of Wang et al. (2015) [45]. For identified QTLs, the name starts with the abbreviation “iq” (“identified QTL”), followed by the experiment and the chromosome (A1–A10, C1–C9). If more than one identified QTL was obtained in the linkage group, a sequence number was added. For example, *iq.HEM.21GY.C3-2* indicates the second identified QTL for Hem on chromosome C3 in the 21GY environment. For unique QTLs, the designations begin with the abbreviation “uq’’ instead of “iq”. For instance, *uq.C3-1* indicates the first unique QTL on the C3 chromosome.

### 4.5. Prediction of Candidate Genes

The prediction of candidate genes was divided into three steps. First, all of the variant loci between the two parents ‘APL01′ and ‘Holly’ across the whole genome were identified based on the results of resequencing [53]; second, using the probe sequences of SNPs on the genetic map, the SNP markers underlining the CIs of the QTLs were mapped to the physical location of the reference genome of the French winter oilseed rape cultivar ‘Darmor-*bzh*’ by Blast (E-value ≤ 1e^−10^) [54], and then all of the SNPs and insertions/deletions (InDels) were obtained on the basis of the results of the first step. Finally, those SNPs and InDels resulting in non-synonymous mutations or shift mutations were targeted to screen candidate genes for the CF component. In addition, candidate genes were further assessed depending on the functional annotation.

## 5. Conclusions

In this study, 49 QTLs related to CF component traits were detected, and four unique QTLs were further integrated. Combined with functional gene annotation and genome sequence variation, five potential candidate genes were identified. Additionally, five low-CF lines have been identified as excellent germplasm resources. These results help us to understand the genetic mechanism regulating CF components in *B. napus* shoots, and to provide a target for reducing CF content.

## Figures and Tables

**Table 1:** Statistical analysis of crude fiber component in *B. napus* shoots.

Trait	Environment	Parent		Mean (%)	Min (%)	Max (%)	CV ^1^ (%)
		APL01	Holly				
NDF	21NJ	46.89	50.12	47.73	28.92	62.95	11.66
	21GY			51.33	40.76	62.39	9.01
	22NJ			51.64	41.79	60.36	6.22
	22GY			46.51	34.19	68.08	12.13
ADF	21NJ	24.95	31.44	29.61	17.69	43.17	14.95
	21GY			35.85	24.83	47.74	12.71
	22NJ			32.06	20.96	42.23	10.81
	22GY			25.37	6.63	33.73	18.30
Hem	21NJ	22.27	16.67	18.08	7.65	32.59	25.07
	21GY			15.48	6.72	32.67	32.94
	22NJ			19.58	11.39	34.63	19.16
	22GY			21.15	10.49	48.99	31.09
Cel	21NJ	15.72	20.11	22.34	13.08	34.00	16.83
	21GY			21.40	4.70	34.76	25.39
	22NJ			10.58	1.39	28.31	47.87
	22GY			11.28	2.61	17.10	20.60
ADL	21NJ	9.24	12.33	7.26	0.81	19.84	51.77
	21GY			14.45	2.31	30.07	39.68
	22NJ			21.49	2.89	36.55	30.15
	22GY			14.21	3.97	22.99	32.65

The codes 22GY, 22NJ, 21GY, and 21NJ are for the following different years and environments: 2022 Guiyang, 2022 Nanjing, 2021 Guiyang, and 2021 Nanjing, respectively. NDF, ADF, Hem, Cel, and ADL represent the traits neutral detergent fiber, acid detergent fiber, hemicellulose, cellulose, and acid detergent lignin, respectively. ^1^ CV is an abbreviation of the coefficient of variation, which was estimated as the ratio of the standard deviation to the mean of all accessions.

**Table 2:** Correlation coefficient of crude fiber component in Guiyang (above diagonal) and Nanjing (below diagonal).

CF Components	NDF	ADF	ADL	Hem	Cel
NDF	-	0.500 **	0.309 **	0.517 **	−0.248 **
ADF	0.604 **	-	0.603 **	−0.481 **	−0.076
ADL	0.238 **	0.489 **	-	−0.280 **	−0.148 *
Hem	0.615 **	−0.257 **	−0.195 **	-	−0.177 *
Cel	0.178 *	0.184 *	−0.764 **	0.34	-

NDF, ADF, Hem, Cel, and ADL represent the traits neutral detergent fiber, acid detergent fiber, hemicellulose, cellulose, and acid detergent lignin, respectively. * Represents significant at *p* = 0.05; ** Represents significant at *p* = 0.01.

**Table 3:** Unique QTLs for crude fiber component detected in four environments.

Unique QTL	CI	Identified QTL	Position	LOD	Additive	PV	Trait	Environment
*uq.C3-1*	18.63–24.3	*iq.HEM.22GY.13*	17.21	2.96	1.66	5.94	Hem	22GY
		*iq.ADL.22NJ.13*	22.81	3.77	−1.91	8.26	ADL	22NJ
*uq.C3-2*	34.95–35.86	*iq.NDF.21GY.13-1*	35.41	4.73	1.6	11.49	NDF	21GY
		*iq.HEM.21GY.13-2*	35.41	5.7	2.02	14.76	Hem	21GY
*uq.C3-3*	42.75–44.16	*iq.NDF.21GY.13-2*	43.21	3.6	1.42	8.94	NDF	21GY
		*iq.HEM.21GY.13-3*	43.71	3.99	1.56	9.25	Hem	21GY
*uq.C7*	48.18–51.63	*iq.ADL.21NJ.17-1*	49.91	4.87	−1.23	9.9	ADL	21NJ
		*iq.HEM.22GY.17*	49.91	2.94	−1.64	5.92	Hem	22GY

NDF, ADF, Hem, Cel, and ADL indicate the traits neutral detergent fiber, acid detergent fiber, hemicellulose, cellulose, and acid detergent lignin, respectively. The codes 22GY, 22NJ, 21GY, and 21NJ are for the following different years and environments: 2022 Guiyang, 2022 Nanjing, 2021 Guiyang, and 2021 Nanjing, respectively.

**Table 4:** Candidate gene information of crude fiber component associated loci in *B. napus* shoots.

Unique QTL	Marker	Physical Position	Genes	Homologue in *A. thaliana*	Function in *A. thaliana*
*uq.C3-1*	Bn-scaff_18936_1p855050~ Bn-scaff_15877_1-p773307	3386108~4492075	*BnaC03g07110D*	*AT5G60920*	Necessary for oriented cell expansion in *Arabidopsis*
			*BnaC03g09000D*	*AT5G18410*	distorted trichomes and exhibits a diffuse actin cytoskeleton
*uq.C3-2*	Bn-scaff_22728_1-p345946 ~Bn-scaff_15975_1-p11640	4578409~4734829	*BnaC03g12990D*	*AT3G61610*	Galactose mutarotase-like superfamily protein
		6166074~6366534			
*uq.C3-3*	Bn-A03-p6522454~ Bn-scaff_18322_1-p903761	5813129~8130878	*BnaC03g12990D*	*AT3G61610*	Galactose mutarotase-like superfamily protein
*uq.C7*	Bn-scaff_16130_1-p293649~ Bn-scaff_16130_1-p659049	28076804~28446612	*BnaC07g21271D*	*AT1G14720*	Member of Glycoside Hydrolase Family 16
			*BnaC07g21371D*	*AT1G14690*	microtubule-associated protein 65-7

**Table 5:** The top ten lines of *B. napus* shoots with the lowest crude fiber component content in different environments.

Environment	Material Name	CF (%)	Average (%)	Material Name	Hem (%)	Average (%)	Material Name	Cel (%)	Average (%)	Material Name	ADL (%)	Average (%)
NJ	AH162	28.92	41.24	AH162	10.14	12.93	AH006	5.86	10.08	AH196	0.81	3.68
	AH172	39.88		AH119	10.23		AH023	6.69		AH072	0.90	
	AH092	42.06		AH079	11.91		AH158	9.86		AH071	1.30	
	AH165	42.06		AH023	12.87		AH165	10.26		AH031	2.87	
	AH034	42.08		AH086	13.44		AH010	10.99		AH162	4.02	
	AH086	42.54		AH153	13.98		AH153	11.27		AH055	4.80	
	AH158	43.11		AH165	14.14		AH122	11.30		AH075	5.08	
	AH055	43.60		AH161	14.16		AH150	11.41		AH076	5.40	
	AH119	44.00		AH066	14.17		AH019	11.54		AH046	5.72	
	AH170	44.18		AH024	14.31		AH159	11.58		AH172	5.89	
GY	AH032	34.19	37.35	AH145	8.64	11.69	AH034	5.84	6.95	AH121	3.13	6.27
	AH018	34.81		AH001	11.55		AH040	6.06		AH021	3.49	
	AH121	36.84		AH010	11.67		AH035	6.54		AH151	6.01	
	AH035	37.12		AH179	11.72		AH046	6.77		AH103	6.07	
	AH037	37.65		AH032	11.73		AH114	6.87		AH104	6.33	
	AH024	38.04		AH161	11.80		AH037	7.27		AH015	7.06	
	AH036	38.06		AH018	12.32		AH036	7.29		AH041	7.34	
	AH045	38.44		AH030	12.32		AH154	7.47		AH107	7.36	
	AH027	39.02		AH027	12.57		AH113	7.68		AH110	7.79	
	AH034	39.30		AH034	12.58		AH045	7.71		AH035	8.10	

GY and NJ are the codes for the following different environments: Guiyang and Nanjing, respectively. NDF, ADF, Hem, Cel, and ADL represent the traits neutral detergent fiber, acid detergent fiber, hemicellulose, cellulose, and acid detergent lignin, respectively.

## Data Availability

All data analyzed during this study are included in this published article and its Supplementary Information Files.

## Supplementary Materials

The following supporting information can be downloaded at: https://www.mdpi.com/article/10.3390/foods12020403/s1, Table S1: QTL results for crude fiber component of *Brassica napus* shoots.
